# Does postnatal care have a role in improving newborn feeding? A study in 15 sub–Saharan African countries

**DOI:** 10.7189/jogh.07.020506

**Published:** 2017-12

**Authors:** Shane M Khan, Ilene S Speizer, Kavita Singh, Gustavo Angeles, Nana AY Twum–Danso, Pierre Barker

**Affiliations:** 1 Data and Analytics, Division of Data, Research and Policy, United Nations Children’s Fund (UNICEF), New York, New York, USA; 2 Department of Maternal and Child Health, Gillings School of Global Public Health, University of North Carolina at Chapel Hill, Chapel Hill, North Carolina, USA; 3 Carolina Population Center, University of North Carolina at Chapel Hill, Chapel Hill, North Carolina, USA

## Abstract

**Background:**

Breastfeeding is known as a key intervention to improve newborn health and survival while prelacteal feeds (liquids other than breastmilk within 3 days of birth) represents a departure from optimal feeding practices. Recent programmatic guidelines from the WHO and UNICEF outline the need to improve newborn feeding and points to postnatal care (PNC) as a potential mechanism to do so. This study examines if PNC and type of PNC provider are associated with key newborn feeding practices: breastfeeding within 1 day and prelacteal feeds.

**Methods:**

We use data from the Demographic and Health Surveys for 15 sub–Saharan African countries to estimate 4 separate pooled, multilevel, logistic regression models to predict the newborn feeding outcomes.

**Findings:**

PNC is significantly associated with increased breastfeeding within 1day (OR = 1.35, *P* < 0.001) but is not associated with PLFs (OR = 1.04, *P* = 0.195). PNC provided by nurses, midwives and untrained health workers is also associated with higher odds of breastfeeding within 1 day of birth (OR = 1.39, *P* < 0.001, (OR = 1.95, *P* < 0.001) while PNC provided by untrained health workers is associated with increased odds of PLFs (OR = 1.20, *P* = 0.017).

**Conclusions:**

PNC delivered through customary care may be an effective strategy to improve the breastfeeding within 1 day but not to discourage PLFs. Further analysis should be done to examine how these variables operate at the country level to produce finer programmatic insight.

Breastfeeding is recognized as a key intervention to improve the health and survival of children and the use of optimal breastfeeding practices such as exclusive breastfeeding is one of the most effective means to reduce undernutrition, an underlying cause of under–five mortality [1]. The World Health Organization (WHO) and the United National Children’s Fund (UNICEF) recommend early initiation of breastfeeding [2] which refers to breastfeeding of a newborn within an hour of birth. Global monitoring efforts by UNICEF also include initiation of breastfeeding within one day of birth which provides additional information on the feeding patterns of newborns and the behaviors of women. Early initiation of breastfeeding has a number of health benefits, one of which is to reduce neonatal mortality [3–5]. The early ingestion of breastmilk can have positive effects on a newborn’s immune systems such as the provision of immunoglobulins and lymphocytes [6–8], priming of the gastrointestinal tract and decreasing the permeability of the tract to pathogens, including HIV [9,10]. Another health benefit of early initiation of breastfeeding is reduced rates of diarrhea among infants, as demonstrated in Egypt and Pakistan [11,12].

Early initiation of breastfeeding is also associated with a number of factors. One such factor is skin–to–skin contact with the mother [13,14], a form of thermal care which is a recommended means to reduce neonatal mortality [15]. Early breastfeeding is also associated with a number of factors related to contact with the health system. For example, in Brazil, early initiation is associated with vaginal delivery as well as other factors such as antenatal guidance on breastfeeding and having a full term pregnancy [16]. Other studies point out that breastfeeding within an hour of birth is less likely to occur when women have caesarian sections, even in the presence of hospital practices that favor breastfeeding [17,18]. In a review article, authors find that higher socio–economic status is associated with lower odds of breastfeeding initiation but this pattern is only seen in developing countries [19].

Prelacteal feeds (PLFs) represent a departure from optimal newborn feeding practices. PLFs are any liquid other than breast milk that is given to the newborn before breastfeeding is established between the mother and newborn. The WHO and UNICEF outline that for successful breastfeeding, PLFs should be avoided and PLFs should not be encouraged unless medically indicated [20]. These feeds usually occur within the first few days of life and are associated with a number of negative health outcomes for the newborn and mother. These include insufficient maternal milk production, newborn diarrhea and reduced length of breastfeeding duration [21,22]. PLFs can also expose newborns to infections through the ingestion of contaminated food and liquids which can act on the GI tract to increase permeability to pathogens, and hence, increase newborn infections [9,11].

A number of studies have shown factors related to PLFs. For example, PLFs are negatively associated with early initiation of breastfeeding (within an hour of birth) [23]. In India, PLFS were associated with lower maternal education among hospital–delivered infants [24]. However, in rural, Western Uganda, more educated women were more prone to provide PLFs to newborns [25]. In low socio–economic settlements in Karachi, Pakistan, PLFs were associated with having a birth attendant [26]. In a national study in Nepal, women without education, who were not working, who had no antenatal care and were first time mothers were more likely to provide PLFs [27]. Both in India and Vietnam, newborns of women with a cesarean section were more likely to ingest PLFs [24,28].

In a recent joint statement, the WHO and UNICEF recommend that all newborns, regardless of place of birth (whether in a facility or not), should receive a basic package of care, including postnatal care which includes the promotion and support of exclusive breastfeeding and the early initiation of breastfeeding [29]. Interventions such as thermal care, hygienic cord care, examination for danger signs and improving parental knowledge of care seeking are also recommended. The evidence on the importance of PNC from developing countries comes mainly from South Asian countries (India, Bangladesh and Pakistan) and are from interventions and trials at sub–national levels (such as districts, villages and communities) [30–32].

Currently, there is a gap in the literature on how interventions such as PNC are associated with newborn feeding practices at the national level, when delivered through usual services of the government and non–governmental sources of care ie, outside of an intervention setting. The literature is especially sparse for sub–Saharan Africa. The only study we found was a small, cross–sectional study in Ethiopia [33] where PNC was associated with increased odds of timely initiation of breastfeeding. Apart from the issue of generalizability of PNC interventions, we currently do not know which type of provider of PNC is best suited to improve the newborn feeding outcomes. The WHO–UNICEF PNC recommendation acknowledges that skilled and unskilled health workers can provide PNC though skilled providers are better suited [29]. However, in the literature on newborn feeding, we find varying opinions on if skilled or unskilled care can improve breastfeeding. In Bangladesh, for example, specially trained peer counselors can improve initiation and duration of exclusive breastfeeding [34]. However, a literature review finds that trained health care workers (physicians, nurses etc.) were found to be a barrier to providing quality information, counseling and care to women on early breastfeeding [19].

The main objective of this paper is to examine the association between PNC within 1 day and two key newborn feeding practices: breastfeeding within 1 day and prelacteal feeds. Given that WHO–UNICEF recommends both skilled and unskilled health workers to provide PNC and that there are mixed results regarding the association of provider type on newborn feeding, we also examine if the type of provider of PNC is important for the two stated outcomes. We use data from nationally representative surveys in 15 sub–Saharan African countries in a pooled, multi–level analysis, controlling for a number of individual and country–level variables. The results of this paper can provide indications on which types of providers are best suited for the delivery of PNC as it relates to newborn feeding.

## METHODS

### Data and variables

Data for this study are from the USAID–supported Demographic and Health Surveys (DHS). DHS surveys collect data from nationally–representative probability samples of households. Households are selected using a two–stage sample design where census enumeration areas are first selected and then a random sample of households is selected in the second stage. Within selected households, all women ages 15–49 are interviewed and provide information on themselves and their children on various health, population and nutrition issues. Women also provide informed consent to the survey prior to the start of questions. All data are anonymized. This analysis focuses on the last birth in the last two years before the surveys for which information on PNC is provided. We include Benin 2011–2012, Burkina Faso 2010, Comoros 2012, Congo Brazzaville 2012, Cote d’Ivoire 2012, Gabon 2012, Guinee 2012, Mali 2012–2013, Namibia 2013, Niger 2012, Nigeria 2013, Sierra Leone 2013, Tanzania 2010, Uganda 2011 and Zimbabwe 2011, based on the availability of comparable data on PNC.

There are two outcome variables. The first is the percentage of newborns who were breastfed within 1 day of birth among all newborns. The second outcome variable is the percentage of newborns who received a PLF ie, a feed that occurs within 3 days of births that is not breastmilk. The measure of PLFs is based on asking the mother if, within the first 3 days after delivery, the newborn was given anything to drink, other than breast milk. This is only asked for newborns who were ever breastfed.

The key independent variable is PNC within 1 day which refers to any check within 1 day to a newborn following birth. The question also provides examples of what a check may entail (checking temperature, cord etc.). We exclude a check by ‘others’ (such as friends or relatives as these are not likely to be medical). Women were also asked, if for the last birth in the 2 years before the survey, what provider or traditional birth attendant performed the check on the newborn’s health. Qualitative work confirms that women are able to tell coherent narratives about the moments around birth and recognize checks on the health of a child [35]. Given that PLFs can occur anytime within 3 days, we attempt to establish PNC preceding PLFs by defining PNC as a check within 1 day of birth instead of 3 days. Both of these outcomes are binary. To investigate if PNC provider is associated with the outcomes, we create a variable for PNC provided by three categories of caregivers: physicians, nurses/midwives/auxiliary midwives and finally, traditional birth attendants/community health workers/other.

In our models, we introduce a number of statistical controls based on the literature, classified as individual–level controls or country–level controls. We include: age of the mother, previous birth interval, parity, caesarian section of birth, use of antenatal care (ANC), receipt of tetanus toxoid vaccination, skilled delivery, educational level of the woman, marital status, media access (regular access to print and mass media), place of residence and a wealth index of household goods and assets (provided in the DHS data files), constructed using Principal Component Analysis of household–level ownership of goods and assets.

We include 4 binary, country–level variables to account for the variation in the supply of PNC. The five country–level variables are: Gross Domestic Product (GDP) per capita (“high” when US$ 1000 or greater per capita or “low” when below US$ 1000 per capita), per capita government expenditure on health (“high” when US$ 100 or greater per capita and “low” when below US$ 100 per capita), number of physicians per 1000 population (“high” when the value is 0.1 or greater and “low” when the value is below 0.1) and finally, the number of nurses per 1000 population (“high” when the value is 1 and greater and “low” when the value is less than 1). Finally, since there are prominent recommendations on newborn feeding practices in areas of high HIV prevalence, we included a dummy variable for HIV prevalence (“high” when 5% or greater and “low” when less than 5%) as an explanatory variable in the models.

### Statistical analysis

We use descriptive statistics and multivariate models to examine the association between the main predictors and the outcomes. First, we describe the sample using frequencies of the variables and then produce cross–tabulations of key variables by the outcome variables using chi–square tests. Finally, we model the outcome variables on the key variables (in separate models), with a number of statistical controls. Univariate analysis is done at the country level to provide an indication of the contribution of each country to overall sample but as the aim of the analysis is cross–country, the remainder of the analysis is done at the aggregate level.

As breastfeeding within 1 day and PLFs are binary outcomes, a logistic regression model can be used, assuming that the error term follows a logistic distribution. However, as we study individual–level data from different countries, this suggest that these data are clustered and as a consequence, a multilevel model may be required (MLM). To verify if MLM is needed, we compared all MLMs to single level logistic regressions using a Likliehood–ratio (LR) test. These results should that the data are clustered at the country level and that MLMs perform better than the single–level logistic regressions. In our models, country–level variance was between 4 to 11 percent. Multilevel models and bivariate table are run without sample weights while univariate are weighted using DHS sample weights provided in datafiles.

## RESULTS

Breastfeeding within a day of birth is high (81 percent) and varies considerably across the countries, ranging from 66 percent in Cote d’Ivoire to 94 percent in Mali (**Table 1**). Levels of prelacteal feeds are lower (39 percent overall), ranging from 11 percent in Namibia to 65 percent in Cote d’Ivoire. PNC is low overall; only 15 percent of the sample received PNC within a day, of which the vast majority was provided by a nurse (12 percent) and only 2 and 1 percent provided by physicians and by traditional birth attendants/community health workers/others (TBA/CHWs/others) respectively. In the sample, about half of the women had 3 or fewer children. Caesarian sections are uncommon (4 percent). More than half of the women had contact with the health system through ANC care (52 percent), receipt of tetanus toxoid (56 percent) and had a skilled delivery (62 percent). The majority of the sample is married, has no education, no regular access to media and about 40 percent is classified into the poorest or second lowest wealth quintiles.

**Table 1:** Weighted distribution of sample for 15 countries

	Benin 2011–2012	Burkina Faso 2010	Comoros 2012	Congo Brazzaville 2012	Cote d’Ivoire 2012	Gabon 2012	Guinee 2012	Mali 2012–2013
**Outcomes**								
Breastfeeding within 1 d of birth:								
Yes	80.9	80.5	76.3	69.8	66.2	70.0	73.1	94.0
No	19.1	19.5	23.7	30.2	33.8	30.0	26.9	6.0
Prelacteal feeding:*								
Yes	18.1	35.9	37.5	36.1	65.6	41.3	59.1	21.1
No	81.9	64.1	62.5	63.9	34.4	58.7	40.9	78.9
**Key variables**								
PNC within 1 d:								
Yes:	20.6	18.0	10.3	15.4	24.8	12.7	16.7	13.5
By Physician	2.0	0.2	1.6	2.7	2.7	1.2	4.4	1.7
By Nurse/Midwife/Aux. midwife	17.4	17.5	8.2	12.6	17.5	11.2	9.5	7.2
By TBA/CHW/Other	1.1	0.2	0.6	0.1	4.6	0.2	2.8	4.5
No	79.4	82.0	89.7	84.6	75.2	87.3	83.3	86.5
**Maternal factors**								
Age of mother:								
15–19	6.2	8.6	8.5	14.0	12.2	14.9	14.3	11.3
20–24	22.1	26.6	22.7	25.8	26.3	25.8	23.1	22.9
25–29	31.8	25.3	24.4	25.8	27.3	24.3	25.4	28.6
30–34	22.1	19.9	23.5	18.1	18.7	18.3	17.1	19.2
35–39	12.0	12.9	15.1	12.6	10.1	11.1	12.8	12.1
40–49	5.7	6.8	5.8	3.8	5.4	5.6	7.3	5.8
Previous birth interval:								
First birth (and twins)	20.8	17.6	22.4	23.7	22.5	27.9	21.2	17.1
<18 months	2.6	1.9	9.2	3.4	3.1	4.8	1.4	3.9
18–23 months	6.5	6.0	12.4	7.3	6.1	8.7	5.7	8.1
24–29 months	13.8	13.2	13.7	12.6	13.1	11.7	9.5	14.4
30–35 months	14.0	17.7	10.7	10.2	13.2	8.1	15.7	14.2
36–47 months (ref)	20.3	23.4	13.6	15.0	16.4	12.0	21.1	20.2
48–53 months	6.2	6.0	4.9	6.1	5.3	4.7	7.2	5.7
54+ months	15.7	14.2	13.1	21.7	20.3	22.1	18.3	16.4
Parity:								
1	20.5	17.5	22.1	23.4	22.1	27.6	21.1	17.0
2–3	38.7	33.8	35.3	42.4	37.3	38.2	33.0	33.4
4–5	24.5	23.3	23.6	22.9	22.8	20.1	23.5	27.1
6+	16.3	25.3	19.0	11.2	17.7	14.1	22.4	22.5
Cesarean section:								
Yes	6.1	2.1	11.4	6.6	3.0	10.6	3.0	3.0
No	93.9	97.9	88.6	93.4	97.0	89.4	97.0	97.0
**Personal illness control factors**								
Antenatal care (4+ with any provider):								
Yes	58.7	32.5	47.6	76.0	42.8	75.6	56.2	41.0
No	41.3	67.5	52.4	24.0	57.2	24.4	43.8	59.0
Tetanus toxoid (2+ during last pregnancy):								
Yes	59.4	70.3	36.2	59.9	52.1	66.5	70.1	36.8
No	40.6	29.7	63.8	40.1	47.9	33.5	29.9	63.2
Skilled delivery:								
Yes	85.6	74.2	85.6	94.1	61.4	91.2	46.2	61.2
No	14.4	25.8	14.4	5.9	38.6	8.8	53.8	38.8
**Socio–economic factors**								
Education of mother:								
None	69.7	83.4	43.3	7.0	62.4	5.8	75.5	81.6
Primary	16.7	10.8	24.9	31.1	26.5	25.9	13.6	9.1
Secondary+	13.6	5.7	31.8	61.9	11.2	68.3	10.9	9.3
Marital status:								
Married/cohabiting	93.6	97.1	94.5	78.3	83.4	70.3	92.3	96.7
Not currently married/cohabiting	6.4	2.9	5.5	21.7	16.6	29.7	7.7	3.3
Media access:								
Yes	22.5	9.2	26.6	25.9	17.0	46.7	17.0	23.7
No	77.5	90.8	73.4	74.1	83.0	53.3	83.0	76.3
Household wealth status:								
Poorest quintile	20.3	20.2	23.0	22.2	24.3	21.3	22.9	20.4
Second quintile	20.5	21.9	20.8	23.0	20.4	21.6	21.4	20.2
Middle quintile	19.4	22.0	21.1	20.2	20.7	22.5	20.7	19.4
Fourth quintile	19.7	21.0	18.5	19.0	18.6	19.3	19.1	22.1
Richest quintile	20.1	14.9	16.6	15.5	15.9	15.2	15.9	17.8
Residence:								
Urban	41.3	17.0	28.4	61.4	38.7	84.3	26.5	20.3
Rural	58.7	83.0	71.6	38.6	61.3	15.7	73.5	79.7
**Country–level characteristics**								
GDP per capita (US$):								
High (1000 per capita and greater)	–	–	–	–	–	–	–	–
Low (less than 1000 per capita)	–	–	–	–	–	–	–	–
Per capita government expenditure on health at average exchange rate (US$):								
High (100 per capita and greater)	–	–	–	–	–	–	–	–
Low (less than 100 per capita)	–	–	–	–	–	–	–	–
No. physicians per 1000 population:								
High (0.1 or greater)	–	–	–	–	–	–	–	–
Low (less than 0.1)	–	–	–	–	–	–	–	–
No. nurses per 1000 population:								
High (1 or greater)	–	–	–	–	–	–	–	–
Low (less than 1)	–	–	–	–	–	–	–	–
HIV prevalence:								
High (5%+)	–	–	–	–	–	–	–	–
Low (<5%)	–	–	–	–	–	–	–	–
**Total**	**5130**	**5988**	**1298**	**3426**	**3039**	**2102**	**2818**	**3965**
								
	**Namibia 2013**	**Niger 2012**	**Nigeria 2013**	**Sierra Leone 2013**	**Tanzania 2010**	**Uganda 2011**	**Zimbabwe 2011**	**All countries**
**Outcomes**								
Breastfeeding within 1 d of birth:								
Yes	89.1	78.6	73.7	89.1	90.5	88.7	91.7	80.1
No	10.9	21.4	26.3	10.9	9.5	11.3	8.3	19.8
Prelacteal feeding:*								
Yes	10.2	49.1	58.4	20.7	30.8	41.1	13.1	39.1
No	89.8	50.9	41.6	79.3	69.2	58.9	86.9	60.9
**Key variables**								
PNC within 1 d:								
Yes:	15.3	10.7	11.4	26.4	1.2	8.8	9.5	14.6
By Physician	5.6	0.2	4.5	1.5	0.1	1.8	1.6	2.3
By Nurse/Midwife/Aux. midwife	9.5	8.8	5.9	21.0	0.9	6.7	7.7	10.9
By TBA/CHW/Other	0.2	1.7	1.0	3.9	0.2	0.3	0.2	1.5
No	84.7	89.3	88.6	73.6	98.8	91.2	90.5	85.4
**Maternal factors**								
Age of mother:								
15–19	10.7	9.6	8.5	13.5	10.2	10.3	12.4	10.4
20–24	25.5	23.1	22.7	23.0	27.1	28.2	31.2	24.5
25–29	25.5	27.4	28.0	26.1	25.4	27.5	27.6	27.1
30–34	20.1	20.8	20.1	18.0	17.5	16.2	16.3	19.3
35–39	12.3	13.0	13.4	12.9	14.2	12.5	9.0	12.6
40–49	5.9	6.1	7.3	6.5	5.5	5.4	3.5	6.1
Previous birth interval:								
First birth (and twins)	32.2	13.6	20.3	22.0	19.9	17.2	29.3	20.7
<18 months	2.4	4.1	4.1	2.5	3.6	6.1	2.4	3.5
18–23 months	4.9	11.3	9.9	6.9	8.0	12.9	3.6	8.1
24–29 months	8.2	20.6	15.7	12.0	16.9	20.5	6.8	14.3
30–35 months	7.7	18.1	15.1	13.9	15.6	13.7	8.7	14.2
36–47 months (ref)	9.8	18.3	18.1	16.7	16.0	15.0	14.9	17.8
48–53 months	4.9	4.3	4.4	5.7	5.0	3.4	5.8	5.2
54+ months	29.8	9.7	12.4	20.4	14.9	11.1	28.5	16.3
Parity:								
1	31.7	13.4	20.1	21.7	19.6	17.1	29.0	20.4
2–3	42.7	27.4	32.3	35.0	35.7	31.5	47.4	35.1
4–5	17.4	24.6	22.6	24.8	23.2	22.4	16.6	23.1
6+	8.2	34.5	25.0	18.6	21.5	29.0	6.9	21.4
Cesarean section:								
Yes	15.7	1.4	2.2	4.0	5.2	5.5	4.5	4.3
No	84.3	98.6	97.8	96.0	94.8	94.5	95.5	95.7
**Personal illness control factors**								
Antenatal care (4+ with any provider):								
Yes	62.0	33.1	51.1	76.0	38.4	46.2	59.2	51.4
No	38.0	66.9	48.9	24.0	61.6	53.8	40.8	48.6
Tetanus toxoid (2+ during last pregnancy):								
Yes	33.9	50.2	48.7	86.7	44.1	52.2	42.8	55.4
No	66.1	49.8	51.3	13.3	55.9	47.8	57.2	44.6
Skilled delivery:								
Yes	89.0	33.4	42.4	62.6	49.7	60.9	64.9	61.6
No	11.0	66.6	57.6	37.4	50.3	39.1	35.1	38.4
**Socio–economic factors**								
Education of mother:								
None	5.6	85.3	47.6	64.7	25.6	12.9	1.1	51.8
Primary	22.5	9.6	18.1	15.3	67.0	63.9	31.3	22.7
Secondary+	71.9	5.1	34.3	20.1	7.4	23.2	67.5	25.5
Marital status:								
Married/cohabiting	44.2	98.3	95.6	84.7	84.0	85.5	87.3	89.3
Not currently married/cohabiting	55.8	1.7	4.4	15.3	16.0	14.5	12.7	10.7
Media access:								
Yes	34.9	7.4	22.2	7.5	18.0	16.0	19.8	19.1
No	65.1	92.6	77.8	92.5	82.0	84.0	80.2	80.9
Household wealth status:								
Poorest quintile	21.3	19.3	23.2	23.0	21.0	22.4	22.2	21.8
Second quintile	22.6	20.5	22.8	21.0	23.9	22.0	21.1	21.7
Middle quintile	21.7	20.8	18.9	21.9	21.7	19.5	19.5	20.4
Fourth quintile	20.0	21.1	18.0	19.1	18.8	18.1	21.2	19.5
Richest quintile	14.4	18.3	17.1	14.9	14.6	18.0	16.0	16.6
Residence:								
Urban	47.5	13.5	35.3	25.7	20.9	14.6	29.3	31.5
Rural	52.5	86.5	64.7	74.3	79.1	85.4	70.7	68.5
**Country–level characteristics**								
GDP per capita (US$):								
High (1000 per capita and greater)								
Low (less than 1000 per capita)	–	–	–	–	–	–	–	37.7
Per capita government expenditure on health at average exchange rate (US$):	–	–	–	–	–	–	–	62.3
High (100 per capita and greater)	–	–	–	–	–	–	–	50.7
Low (less than 100 per capita)	–	–	–	–	–	–	–	49.3
No. physicians per 1000 population:								
High (0.1 or greater)	–	–	–	–	–	–	–	43.9
Low (less than 0.1)	–	–	–	–	–	–	–	56.1
No. nurses per 1000 population:								
High (1 or greater)	–	–	–	–	–	–	–	36.2
Low (less than 1)	–	–	–	–	–	–	–	63.8
HIV prevalence:								
High (5%+)	–	–	–	–	–	–	–	17.6
Low (<5%)	–	–	–	–	–	–	–	82.4
**Total**	1947	5143	12473	4820	3266	3092	2448	60956

PNC – postnatal care, TBA – Traditional Birth Attendant, CHW – Community Health Worker

*Denominator is ever–breast fed newborns.

**Figure 1:**
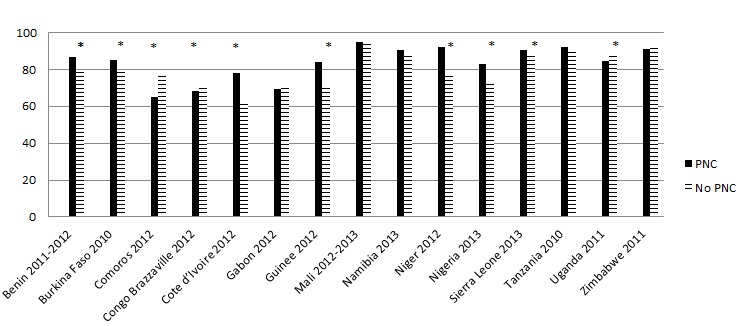
Percentage of newborns breastfed within 1 day of birth by post–natal care within 1 day. Asterisk indicates *P* < 0.05.

**Figure 2:**
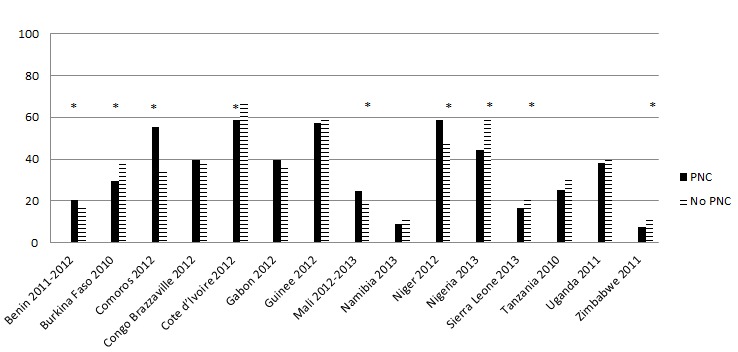
Among ever breast–fed newborns, percentage who had a prelacteal feed by post–natal care within 1 day of birth. Asterisk indicates *P* < 0.05.

In 7 of the 15 countries, newborns who receive PNC were more likely to be breastfed within 1 day compared with newborns who did not receive PNC but in several countries (eg, Comoros, Congo (Brazzaville), Uganda), the opposite occurs (**Figure 1**). **Figure 2** shows that while overall newborns receiving PNC are significantly less likely to receive a prelacteal feed, patterns by country vary considerably; 5 countries show a statistically significant relationship but 4 show the opposite pattern.

In the bivariate analysis, newborns receiving PNC within 1 day are significantly more likely to initiate breastfeeding within a day and less likely to receive a prelacteal feed (**Table 2**). Women receiving antenatal care, tetanus toxoid and skilled delivery are significantly more likely to breastfeed within a day and less likely to provide a prelacteal feed to the newborn. A caesarian birth is significantly associated with breastfeeding within 1 day but not with PLFs. Women with no education are less likely to breastfeed early and more likely to provide a prelacteal feed. While household wealth is positively associated with breastfeeding within 1 day, the association is negative with prelacetal feeds. Women in urban areas are more likely than rural women to initiate breastfeeding within 1 day and less likely to give a prelacetal feed. Bivariate analysis of the country–level variables also shows lower levels of GDP, expenditures, and physician and nurse density are associated with greater initiation of breastfeeding within 1 day and lower levels of prelacteal feeds. In countries with higher HIV prevalence, breastfeeding within 1 day is higher and prelacteal feeds are lower.

**Table 2:** Percentage of all newborns breastfed within 1 day and percentage of newborns receiving prelacteal feeds among ever breastfed newborns, by key characteristics (unweighted), 15 countries

	All newborns		Ever breastfed newborns	
	**Breastfeeding within:**		**Prelacteal feed**	
	**1 day**	***P***		***P***
**Key dependent variables**				
PNC within 1 day:				
Yes	84.2	<0.001	35.3	<0.001
No	79.8		39.3	
**Maternal factors**				
Age of mother:				
15–19	76.3	<0.001	42.5	<0.001
20–24	80.1		38.9	
25–29	81.9		37.0	
30–34	81.4		37.7	
35–39	80.8		38.6	
40–45	80.4		40.7	
45–49	79.9		45.3	
Previous birth interval:				
First birth (and twins)	81.8	<0.001	38.7	<0.001
<18 months	76.8		39.6	
18–23 months	78.2		41.6	
24–29 months	80.5		41.6	
30–35 months	82.1		40.7	
36–47 months	81.8		39.7	
48–53 months	81.5		35.7	
54+ months	81.5		33.7	
Parity:				
1	77.0	<0.001	39.6	<0.001
2–3	81.9		35.6	
4–5	82.2		38.2	
6+	79.9		43.2	
Cesarean section:				
Yes	62.4	<0.001	38.3	0.710
No	81.3		38.7	
Breastfed within 1 hour:				
Yes	–		28.0	<0.001
No	–		47.3	
**Personal illness control factors**				
Antenatal care (4+ with any provider):				
Yes	82.0	<0.001	33.9	<0.001
No	78.9		43.8	
Tetanus toxoid (2+ during last pregnancy):				
Yes	81.5	<0.001	35.5	<0.001
No	79.2		42.7	
Skilled delivery:				
Yes	82.8	<0.001	30.9	<0.001
No	76.7		51.4	
**Socio–economic factors**				
Education of mother:				
None	79.3	<0.001	42.7	<0.001
Primary	82.4		37.1	
Secondary+	81.2		32.2	
Marital status:				
Married/cohabiting	80.7	0.001	39.4	<0.001
Not currently married/cohabiting	79.0		33.2	
Media access:				
Yes	81.6	0.001	33.6	<0.001
No	80.3		39.8	
Household wealth status:				
Poorest quintile	77.2	<0.001	43.2	<0.001
Second quintile	79.2		40.8	
Middle quintile	82.1		38.1	
Fourth quintile	82.3		35.7	
Richest quintile	83.1		33.3	
Residence:				
Urban	81.7	<0.001	34.2	<0.001
Rural	80.0		40.6	
**Country characteristics**				
GDP per capita (US$):				
High (1000+ per capita)	74.0	<0.001	49.7	<0.001
Low (<1000 per capita)	84.7		31.6	
Per capita government expenditure on health at average exchange rate (US$):				
High (100+ per capita)	77.5	<0.001	44.3	<0.001
Low (<100 per capita)	83.7		32.7	
No. physicians per 1000 population.				<0.001
High (0.1+)	75.7	<0.001	50.6	
Low (<0.1)	84.3		29.2	
No. nurses per 1000 population:				
High (1+)	79.1	<0.001	44.1	<0.001
Low (<1)	81.4		35.6	
HIV prevalence:				
High (5%+)	90.2	<0.001	24.9	<0.001
Low (<5%)	78.5		41.6	
**Total**	61018		59309	

PNC – postnatal care

**Table 3** shows that after controlling for individual and country–level variables, PNC within 1 day is significantly associated with higher odds of breastfeeding within 1 day (OR = 1.35, 95% CI 1.27–1.44). The odds of breastfeeding within 1 day are significantly lower for women who had a caesarian section compared with those that did not have a caesarian section (OR = 0.26, 95% CI 0.23–0.28). Many of the variables related to contact with the health care system that are significant at the bivariate level are also significant in the multilevel model. These include ANC (OR = 1.07, 95% CI 1.02–1.12), tetanus coverage (OR = 1.10, 95% CI 1.05–1.15) and skilled delivery (OR = 1.48, 95% CI 1.40–1.56). Several socio–economic variables are significantly associated with breastfeeding within 1 day. Compared to women with no education, women with primary education are significantly more likely to initiate breastfeeding within 1 day (OR = 1.10, 95% CI 1.04–1.17) though the association with secondary or higher education is not significant (OR = 1.06, 95% CI 0.99–1.14). Women in rural areas are significantly less likely to initiate breastfeeding within a day than those in urban areas (OR = 0.92, 95% CI 0.87–0.98). Of the country–level controls in the model, higher HIV prevalence is associated with increased odds of breastfeeding within 1 day (OR = 2.13, 95% CI 1.19–3.82).

**Table 3:** Multilevel logistic regression for breastfeeding within 1 d among all newborns and prelacteal feeds among ever breastfed newborns, 15 countries

	All newborns, breastfeeding within 1 d									Among ever breastfed newborns, prelacteal feeds							
	**Model 1**	***P***	**95% CI**		**Model 2**	***P***	**95% CI**		**Model 3**		***P***	**95% CI**		**Model 4**	***P***	**95% CI**	
*Fixed Effects*																	
**Key variables**																	
PNC within 1 d:																	
Yes	1.35	<0.001	1.27	1.44	–	–	–	–	1.04		0.195	0.98	1.09	–	–	–	–
No	1.00	–	–	–	–	–	–	–	1.00		–	–	–	–	–	–	–
Provider of PNC within 1 d:																	
By Physician	–	–	–	–	0.93	0.269	0.81	1.06	–		–	–	–	0.94	0.343	0.83	1.07
By Nurse/Midwife	–	–	–	–	1.39	<0.001	1.29	1.50	–		–	–	–	1.03	0.315	0.97	1.09
By TBA/CHW/Other	–	–	–	–	1.95	<0.001	1.60	2.36	–		–	–	–	1.20	0.017	1.03	1.39
No	–	–	–	–	1.00	–	–	–	–		–	–	–	1.00	–	–	–
**Maternal factors**																	
Age of mother:																	
15–19	1.00	–	–	–	1.00	–	–	–	1.00		–	–	–	1.00	–	–	–
20–24	1.08	0.049	1.00	1.17	1.09	0.043	1.00	1.17	0.93		0.038	0.86	1.00	0.93	0.040	0.86	1.00
25–29	1.15	0.002	1.05	1.26	1.16	0.001	1.06	1.26	0.84		<0.001	0.78	0.91	0.85	<0.001	0.78	0.92
30–34	1.13	0.020	1.02	1.25	1.14	0.014	1.03	1.26	0.85		<0.001	0.77	0.93	0.85	<0.001	0.77	0.93
35–39	1.12	0.050	1.00	1.26	1.13	0.038	1.01	1.27	0.85		0.002	0.77	0.94	0.85	0.002	0.77	0.94
40–49	1.16	0.027	1.02	1.33	1.17	0.020	1.03	1.34	0.91		0.117	0.81	1.02	0.91	0.127	0.81	1.03
Previous birth interval:																	
First birth (and twins)	0.35	<0.001	0.24	0.49	0.35	<0.001	0.24	0.50	1.19		0.374	0.81	1.77	1.20	0.374	0.81	1.77
<18 months	0.78	<0.001	0.69	0.88	0.78	<0.001	0.69	0.88	1.07		0.181	0.97	1.19	1.07	0.185	0.97	1.19
18–23 months	0.92	0.070	0.84	1.01	0.92	0.069	0.84	1.01	1.04		0.332	0.96	1.12	1.04	0.332	0.96	1.12
24–29 months	1.01	0.733	0.94	1.09	1.01	0.721	0.94	1.09	1.03		0.399	0.96	1.10	1.03	0.400	0.96	1.10
30–35 months	1.01	0.805	0.94	1.09	1.01	0.813	0.94	1.09	0.98		0.624	0.92	1.05	0.98	0.623	0.92	1.05
36–47 months (ref)	1.00	–	–	–	1.00	–	–	–	1.00		–	–	–	1.00	–	–	–
48–53 months	0.93	0.190	0.84	1.04	0.93	0.194	0.84	1.04	0.97		0.457	0.88	1.06	0.97	0.455	0.88	1.06
54+ months	0.89	0.003	0.83	0.96	0.89	0.003	0.83	0.96	0.96		0.231	0.90	1.03	0.96	0.230	0.90	1.03
Parity:																	
1	1.00	–	–	–	1.00	–	–	–	1.00		–	–	–	1.00	–	–	–
2–3	0.48	<0.001	0.34	0.68	0.48	<0.001	0.34	0.69	0.97		0.868	0.65	1.43	0.97	0.869	0.65	1.43
4–5	0.49	<0.001	0.34	0.70	0.49	<0.001	0.34	0.71	1.03		0.889	0.69	1.53	1.03	0.891	0.69	1.53
6+	0.45	<0.001	0.32	0.65	0.45	<0.001	0.32	0.65	1.05		0.794	0.71	1.57	1.05	0.797	0.71	1.57
Cesarean section																	
Yes	0.26	<0.001	0.23	0.28	0.26	<0.001	0.24	0.29	1.60		<0.001	1.46	1.76	1.61	<0.001	1.47	1.77
No					1.00	–	–	–	1.00		–	–	–	1.00	–	–	–
Breastfed within 1 h:																	
Yes	–	–	–	–	–	–	–	–	0.57		<0.001	0.55	0.59	0.57	<0.001	0.55	0.59
No	–	–	–	–	–	–	–	–	1.00		–	–	–	1.00	–	–	–
**Personal illness control factors**																	
Antenatal care (4+ with any provider):																	
Yes	1.07	0.009	1.02	1.12	1.07	0.008	1.02	1.12	0.90		<0.001	0.87	0.94	0.90	<0.001	0.87	0.94
No	1.00	–	–	–	1.00	–	–	–	1.00		–	–	–	1.00	–	–	–
Tetanus toxoid (2+ during last pregnancy):																	
Yes	1.10	<0.001	1.05	1.15	1.10	<0.001	1.05	1.15	0.87		<0.001	0.83	0.90	0.87	<0.001	0.83	0.90
No	1.00	–	–	–	1.00	–	–	–	1.00		–	–	–	1.00	–	–	–
Skilled delivery:																	
Yes	1.48	<0.001	1.40	1.56	1.50	<0.001	1.42	1.59	0.58		<0.001	0.56	0.61	0.59	<0.001	0.56	0.62
No	1.00	–	–	–	1.00	–	–	–	1.00		–	–	–	1.00	–	–	–
**Socio–economic factors**																	
Education of mother:																	
None	1.00	–	–	–	1.00	–	–	–	1.00		–	–	–	1.00	–	–	–
Primary	1.10	0.002	1.04	1.17	1.10	0.002	1.04	1.17	0.86		<0.001	0.82	0.91	0.86	<0.001	0.82	0.91
Secondary+	1.06	0.081	0.99	1.14	1.07	0.061	1.00	1.15	0.78		<0.001	0.73	0.83	0.78	<0.001	0.73	0.83
Marital status:																	
Married/cohabiting	1.13	0.001	1.05	1.21	1.13	0.001	1.05	1.21	1.02		0.624	0.95	1.09	1.02	0.622	0.95	1.09
Not currently married/cohabiting	1.00	–	–	–	1.00	–	–	–	1.00		–	–	–	1.00	–	–	–
Media access:																	
Yes	0.96	0.221	0.90	1.02	0.96	0.260	0.91	1.03	0.99		0.777	0.94	1.05	0.99	0.796	0.94	1.05
No	1.00	–	–	–	1.00	–	–	–	1.00		–	–	–	1.00	–	–	–
Household wealth status:																	
Poorest quintile	1.00	–	–	–	1.00	–	–	–	1.00		–	–	–	1.00	–	–	–
Second quintile	1.05	0.084	0.99	1.12	1.05	0.115	0.99	1.11	0.98		0.418	0.93	1.03	0.98	0.386	0.93	1.03
Middle quintile	1.19	<0.001	1.12	1.28	1.19	<0.001	1.11	1.27	0.95		0.116	0.90	1.01	0.95	0.102	0.90	1.01
Fourth quintile	1.09	0.023	1.01	1.17	1.08	0.030	1.01	1.17	1.01		0.836	0.94	1.07	1.01	0.866	0.94	1.07
Richest quintile	1.10	0.040	1.00	1.21	1.11	0.031	1.01	1.22	1.04		0.309	0.96	1.13	1.04	0.296	0.96	1.13
Residence:																	
Urban	1.00	–	–	–	1.00	–	–	–	1.00		–	–	–	1.00	–	–	–
Rural	0.92	0.006	0.87	0.98	0.92	0.004	0.86	0.97	1.04		0.125	0.99	1.10	1.04	0.143	0.99	1.09
**Country characteristics**																	
GDP per capita (US$):																	
High (1000+ per capita)	0.60	0.137	0.30	1.18	0.60	0.138	0.30	1.18	1.14		0.765	0.48	2.68	1.14	0.762	0.48	2.69
Low (<1000 per capita)	1.00	–	–	–	1.00	–	–	–	1.00		–	–	–	1.00	–	–	–
Per capita government expenditure on health at average exchange rate (US$):																	
High (100+ per capita)	1.08	0.800	0.58	2.03	1.08	0.819	0.57	2.02	1.11		0.803	0.50	2.45	1.10	0.809	0.50	2.44
Low (<100 per capita)	1.00	–	–	–	1.00	–	–	–	1.00		–	–	–	1.00	–	–	–
No. physicians per 1000 population:																	
High (0.1+)	0.62	0.052	0.38	1.00	0.62	0.054	0.38	1.01	2.26		0.009	1.22	4.17	2.26	0.009	1.22	4.18
Low (<0.1)	1.00	–	–	–	1.00	–	–	–	1.00		–	–	–	1.00	–	–	–
No. nurses per 1000 population:																	
High (1+)	1.36	0.348	0.72	2.57	1.37	0.338	0.72	2.59	0.63		0.266	0.28	1.42	0.63	0.269	0.28	1.42
Low (<1)	1.00	–	–	–	1.00	–	–	–	1.00		–	–	–	1.00	–	–	–
HIV prevalence:																	
High (5%+)	2.13	0.011	1.19	3.82	2.14	0.011	1.19	3.83	0.60		0.168	0.29	1.24	0.60	0.171	0.29	1.25
Low (<5%)	1.00	–	–	–	1.00	–	–	–	1.00		–	–	–	1.00	–	–	–
																
*Random effects*																	
Country–level variance (SE):	0.147(0.055)				0.147(.055)				0.237(0.09)					0.238(0.09)			
Log–likelihood	–28 043.57				–28 021.71				–34 632.369					–34 629.4			
AIC	56 159.13				56 119.41				69 338.7					69 336.8			
Log–likelihood ratio test (Chi–square)	715.1*				712.06*				1803.4*					1808.2*			
**Total**	**61 018**				**61 018**				**59 309**					**59 309**			

PNC – postnatal care, TBA – Traditional Birth Attendant, CHW – Community Health Worker

*P < 0.01.

Model 2 shows that the provider of PNC is significantly associated with breastfeeding within 1 day. PNC from physicians is not associated with breastfeeding within 1 day but PNC provided by nurses/midwives/auxiliary midwives and TBA/CHW/others is associated with higher odds of breastfeeding within 1 day (nurses/midwives/aux. midwives OR = 1.39, 95% CI 1.29–1.50, TBA/CHW/others OR = 1.95, CI 1.60–2.36).

**Table 3** shows that after controlling for individual and country–level variables, PNC within 1 day is not significantly associated with prelacteal feeds (OR = 1.04, 95% CI 0.98–1.09). Age is significantly associated with the outcome in the model with older women tending to have lower odds of providing prelacteal feeds to newborn while birth spacing and parity were not associated with prelacteal feeds. Newborns who had a Caesarian section delivery are significantly more likely to have PLFs (OR = 1.60, 95% CI 1.46–1.76). Contact with the health care system through ANC, tetanus toxoid vaccination and skilled delivery are significantly associated with lower odds of prelacteal feeds (see **Table 3**). For example, skilled delivery is associated with a 42% reduction in odds of prelacteal feeding (OR = 0.58, 95% CI 0.56–0.61). Education shows a clear gradient with prelacteal feeds; as the educational level of the woman increases, the odds of prelacteal feeding decreases (see **Table 3**). Of the country–level characteristics, only the density of physicians is significantly associated with prelacteal feeds in the models: higher density of physicians is associated with higher odds of prelacteal feeds (OR = 2.26, CI 1.22–4.17). In model 4 of **Table 3**, the type of provider of PNC is not associated with prelacteal feeds. Other results remain similar to model 2 of the second panel of **Table 3**.

## DISCUSSION

PNC is one of the current strategies recommended for scale–up and implementation in many developing countries to improve health outcomes for newborns and mothers. While several trials and intervention studies show that PNC can improve newborn feeding patterns [30-32], this is the first study to demonstrate this association using national–level data for multiple countries in sub–Saharan Africa.

The major findings are that PNC is associated with breastfeeding within 1 day though not with prelacteal feeds. These findings are important as they suggest that PNC when delivered through customary care (as opposed to intervention and trial conditions) can be a useful strategy to improve breastfeeding (within 1 day) but not to reduce PLFs. These findings highlight the need to strengthen clinical practice so that providers of PNC can move beyond promoting timely initiation of breastfeeding to providing more emphasis on the avoidance of PLFs, which by definition would improve exclusive breastfeeding rates in these countries.

Our findings also indicate that both trained medical personal (nurses, midwives and auxiliary midwives) and untrained providers of PNC are associated with increased odds of breastfeeding within 1 day though the type of provider of PNC is not associated with PLFs. Given that all of the countries that we studied are developing countries, use of untrained persons for this type of intervention may be a useful implementation approach as the promotion of optimal newborn feeding does not require high levels of specialized training

A third important finding from this study is that, with the exception of caesarian section, contact with the formal health care system is associated with improved newborn feeding practices. This is seen in other studies eg, Nepal [27] and India [24]. This underscores the utility of the continuum of care and reinforces the need to implement around this framework. Delivery mode by caesarian section, however, is associated with poorer newborn feeding outcomes, a finding that is reflected in a number of other studies [16,24,28,36,37], even in the presence of baby–friendly policies [17].

Our study has a number of limitations. DHS data do not include any information on what procedures were done during a check and therefore cannot control for content of care. We also use cross–sectional data where PNC was not randomly assigned to individuals. As such, we are not able to provide causal linkages between PNC and the outcomes though we are able to examine associations. One of the more studied variables on breastfeeding initiation is breastfeeding within 1 hour of birth. With our data, we could study the association of PNC within an hour and breastfeeding within the same time period. However, we considered that a short time period of 1 hour does not provide sufficient time for PNC to be provided (given that in these settings, even PNC within 1 day is low). The literature also identifies a number of additional factors that predict early initiation of breastfeeding and PLFs which were not available for analysis. For example, intention to breastfeed [38] is an important predictor of initiation and duration of breastfeeding but was not available in DHS data. Dealing with sample weights is a challenge for analysis of this kind. Different countries contribute varying proportions of the overall sample and do not reflect the relative population size of the country. Appropriate sample weight can be constructed though the sample weights must be de–normalized. However, the appropriate sampling fraction for each country and their population sizes used to create these weights are not publicly available.

Despite these limitations, our findings are consistent with trials and intervention studies, and overall, PNC policy and practice can be further tailored to reduce PLFs rates. Further research at a country–level is needed to understand if the results of this aggregate, multi–country study are reflected within each of these countries.

## References

[1] Bhutta ZA, Ahmed T, Black RE, Cousens S, Dewey K, Giugliani E (2008). What works? Interventions for maternal and child undernutrition and survival.. Lancet.

[2] World Health Organization, United Nations Children’s Fund. Global strategy for infant and young child feeding. Geneva: World Health Organization, 2003.

[3] Edmond KM, Zandoh C, Quigley MA, Amenga-Etego S, Owusu-Agyei S, Kirkwood BR (2006). Delayed breastfeeding initiation increases risk of neonatal mortality.. Pediatrics.

[4] Huffman SL, Zehner ER, Victora C (2001). Can improvements in breast-feeding practices reduce neonatal mortality in developing countries?. Midwifery.

[5] Mullany LC, Katz J, Li YM, Khatry SK, LeClerq SC, Darmstadt GL (2008). Breast-feeding patterns, time to initiation, and mortality risk among newborns in southern Nepal.. J Nutr.

[6] Goldman AS (1993). The immune system of human milk: antimicrobial, antiinflammatory and immunomodulating properties.. Pediatr Infect Dis J.

[7] Goldman AS, Garza C, Nichols BL, Goldblum RM (1982). Immunologic factors in human milk during the first year of lactation.. J Pediatr.

[8] Brandtzaeg P (2003). Mucosal immunity: integration between mother and the breast-fed infant.. Vaccine.

[9] Goldman AS (2000). Modulation of the gastrointestinal tract of infants by human milk. Interfaces and interactions. An evolutionary perspective.. J Nutr.

[10] Rollins NC, Filteau SM, Coutsoudis A, Tomkins AM (2001). Feeding mode, intestinal permeability, and neopterin excretion: a longitudinal study in infants of HIV-infected South African women.. J Acquir Immune Defic Syndr.

[11] Badruddin SH, Islam A, Hendricks KM, Bhutta ZA, Shaikh S, Snyder JD (1991). Dietary risk factors associated with acute and persistent diarrhea in children in Karachi, Pakistan.. Am J Clin Nutr.

[12] Clemens J, Elyazeed RA, Rao M, Savarino S, Morsy BZ, Kim Y (1999). Early initiation of breastfeeding and the risk of infant diarrhea in rural Egypt.. Pediatrics.

[13] Sinusas K, Gagliardi A (2001). Initial management of breastfeeding.. Am Fam Physician.

[14] World Health Organization. Pregnancy, childbirth, postpartum and newborn care: A guide for essential practice. Geneva: World Health Organization, 2003. 26561684

[15] Darmstadt GL, Bhutta ZA, Cousens S, Adam T, Walker N, de Bernis L (2005). Evidence-based, cost-effective interventions: how many newborn babies can we save?. Lancet.

[16] Vieira TO, Vieira GO, Giugliani ERJ, Mendes CM, Martins CC, Silva LR (2010). Determinants of breastfeeding initiation within the first hour of life in a Brazilian population: cross-sectional study.. BMC Public Health.

[17] Rowe-Murray HJ, Fisher JRW (2002). Baby friendly hospital practices: cesarean section is a persistent barrier to early initiation of breastfeeding.. Birth.

[18] Boccolini CS, de Carvalho ML, de Oliveira MIC, Leal Mdo, Carvalho (2008). Factors that affect time between birth and first breastfeeding.. Cad Saude Publica.

[19] Dennis C-L (2002). Breastfeeding initiation and duration: a 1990-2000 literature review.. J Obstet Gynecol Neonatal Nurs.

[20] World Health Organization. Evidence for the ten steps to successful breastfeeding. Geneva: World Health Organization, 1998.

[21] Hossain MM, Radwan MM, Arafa SA, Habib M, DuPont HL (1992). Prelacteal infant feeding practices in rural Egypt.. J Trop Pediatr.

[22] Lakati AS, Makokha OA, Binns CW, Kombe Y (2010). The effect of pre-lacteal feeding on full breastfeeding in Nairobi, Kenya.. East Afr J Public Health.

[23] El-Gilany AH, Sarraf B, Al-Wehady A (2012). Factors associated with timely initiation of breastfeeding in Al-Hassa province, Saudi Arabia.. East Mediterr Health J.

[24] Patel A, Banerjee A, Kaletwad A (2013). Factors associated with prelacteal feeding and timely initiation of breastfeeding in hospital-delivered infants in India.. J Hum Lact.

[25] Wamani H Astrřm AN Peterson STylleskär T Tumwine JK Infant and young child feeding in western Uganda: knowledge, practices and socio-economic correlates. J Trop Pediatr 2005 51 356 61 10.1093/tropej/fmi048 15947011

[26] Fikree FF, Ali TS, Durocher JM, Rahbar MH (2005). Newborn care practices in low socioeconomic settlements of Karachi, Pakistan.. Soc Sci Med.

[27] Khanal V, Adhikari M, Sauer K, Zhao Y (2013). Factors associated with the introduction of prelacteal feeds in Nepal: findings from the Nepal Demographic and Health Survey 2011.. Int Breastfeed J.

[28] Nguyen PH, Keithly SC, Nguyen NT (2013). Prelacteal feeding practices in Vietnam: challenges and associated factors.. BMC Public Health.

[29] World Health Organization, United Nations Children’s Fund. Home visits for the newborn child. Geneva: World Health Organization, 2009.

[30] Bhutta ZA, Memon ZA, Soofi S, Salat MS, Cousens S, Martines J (2008). Implementing community-based perinatal care: results from a pilot study in rural Pakistan.. Bull World Health Organ.

[31] Kumar V, Mohanty S, Kumar A, Misra RP, Santosham M, Awasthi S (2008). Effect of community-based behaviour change management on neonatal mortality in Shivgarh, Uttar Pradesh, India: a cluster-randomised controlled trial.. Lancet.

[32] Baqui AH, Ahmed S, El Arifeen S, Darmstadt GL, Rosecrans AM, Mannan I (2009). Effect of timing of first postnatal care home visit on neonatal mortality in Bangladesh: a observational cohort study.. BMJ.

[33] Setegn T, Gerbaba M, Belachew T (2011). Determinants of timely initiation of breastfeeding among mothers in Goba Woreda, South East Ethiopia: a cross sectional study.. BMC Public Health.

[34] Haider R, Ashworth A, Kabir I, Huttly SR (2000). Effect of community-based peer counsellors on exclusive breastfeeding practices in Dhaka, Bangladesh: a randomised controlled trial. Lancet.

[35] Yoder P. Stanley, Mikey Risato, Riad Mahmud, Alfredo Fort, Fazlur Rahman, Avril Armstrong, and Sayed Rubayet. Women's recall of delivery and neonatal care in Bangladesh and Malawi: A study of terms, concepts, and survey questions. DHS Qualitative Research Studies No. 17. Calverton, Maryland, USA: ICF Macro. 2010. Available: http://dhsprogram.com/pubs/pdf/QRS17/QRS17.pdf. Accessed: 1 August 2017.

[36] Chien L-Y, Tai C-J (2007). Effect of delivery method and timing of breastfeeding initiation on breastfeeding outcomes in Taiwan.. Birth.

[37] El-Gilany A-H, Abdel-Hady DM (2014). Newborn first feed and prelacteal feeds in Mansoura, Egypt.. BioMed Res Int.

[38] Donath SM, Amir LH, ALSPAC Study Team (2003). The relationship between prenatal infant feeding intention and initiation and duration of breastfeeding: a cohort study.. Acta Paediatr.

